# Supplementary material for: Generating kinetic environments to study dynamic cellular processes in single cells
Source: Sci Rep. 2019 Jul 12;9:10129. doi: 10.1038/s41598-019-46438-8 (PMC6625993; doi:10.1038/s41598-019-46438-8)
Supplement: Supplementary file 1 — Supplementary Information for Generating kinetic environments to study dynamic cellular processes in single cells [file 41598_2019_46438_MOESM1_ESM.pdf]

Supplementary Information:

Generating kinetic environments to study dynamic cellular processes in single cells

Authors

Alexander Thiemicke[1*], Hossein Jashnsaz[1*], Guoliang Li[1], Gregor Neuert[1-3]

*equally contributing

Affiliations

[1]Department of Molecular Physiology and Biophysics, School of Medicine, Vanderbilt University, Nashville, TN, 37232, USA.

[2]Department of Biomedical Engineering, School of Engineering, Vanderbilt University, Nashville, TN, 37232, USA.

[3]Department of Pharmacology, School of Medicine, Vanderbilt University, Nashville, TN, 37232, USA.

corresponding author: Gregor Neuert (gregor.neuert@vanderbilt.edu)

Supplementary Figure 1 | A diagram illustration of algorithm to compute the pump profiles.

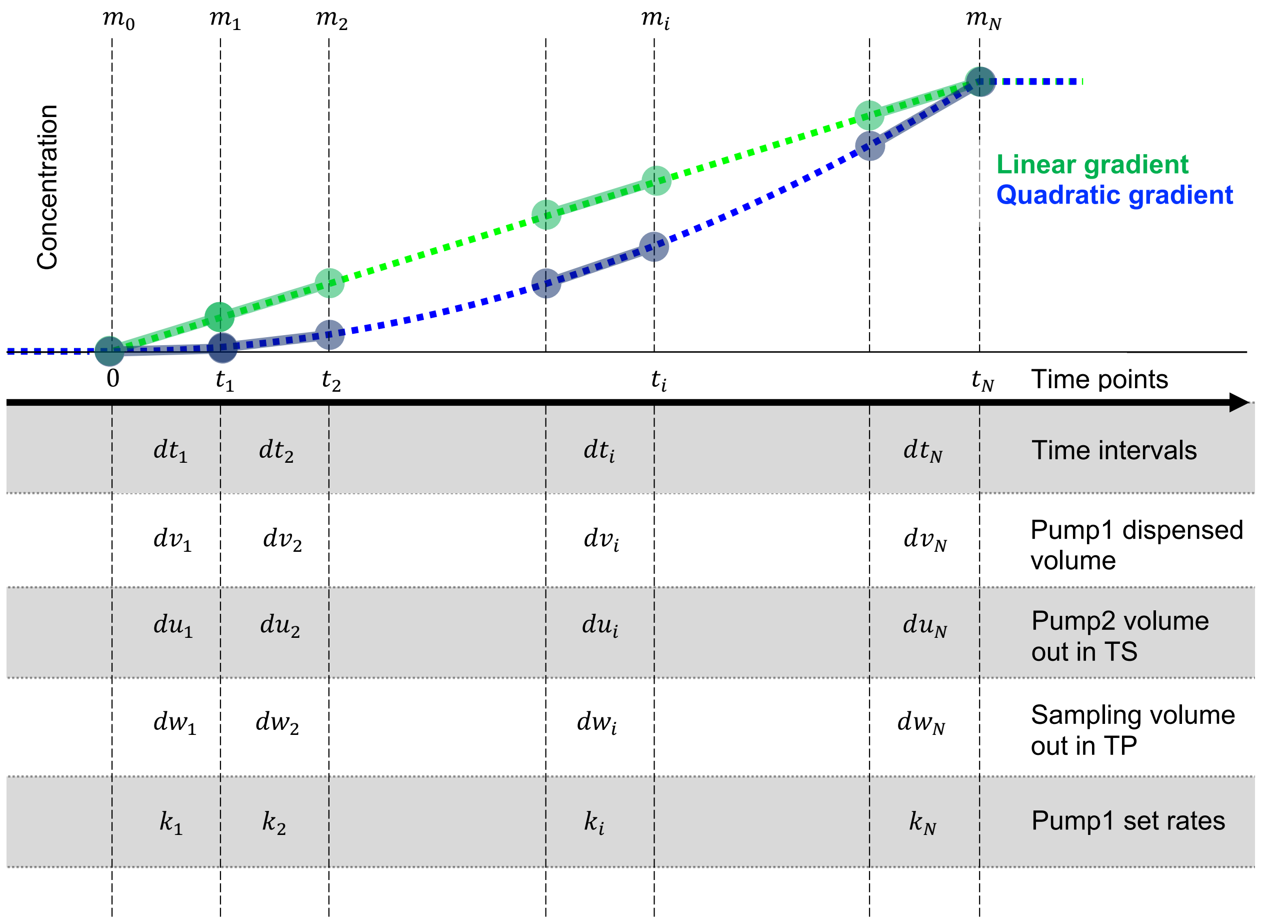

We calculate the stimulus concentration for any profile over discrete time points set by programmable pump by combining several short segments with linear concentration profiles. During each interval, we increase (**Supplementary Figure 2**) or decrease (**Supplementary Figure 3**) the concentration linearly with a fixed rate to achieve increasing or decreasing kinetics of any shape over time. For linear gradient kinetics, the rates are fixed during all intervals ($k_1 = k_2 = \cdots k_N$). By changing the rate from one interval to the next, any arbitrary profile over the whole treatment time can be generated ($k_1 \neq k_2 \neq \cdots k_N$). During each interval (dt_i), stimulus is delivered continually over time by adding appropriate amount (dv_i) of concentrated stimulus. The profiles are corrected for the added (dv_i) and removal (du_i and dw_i) volumes therefore change in stimulus concentration.

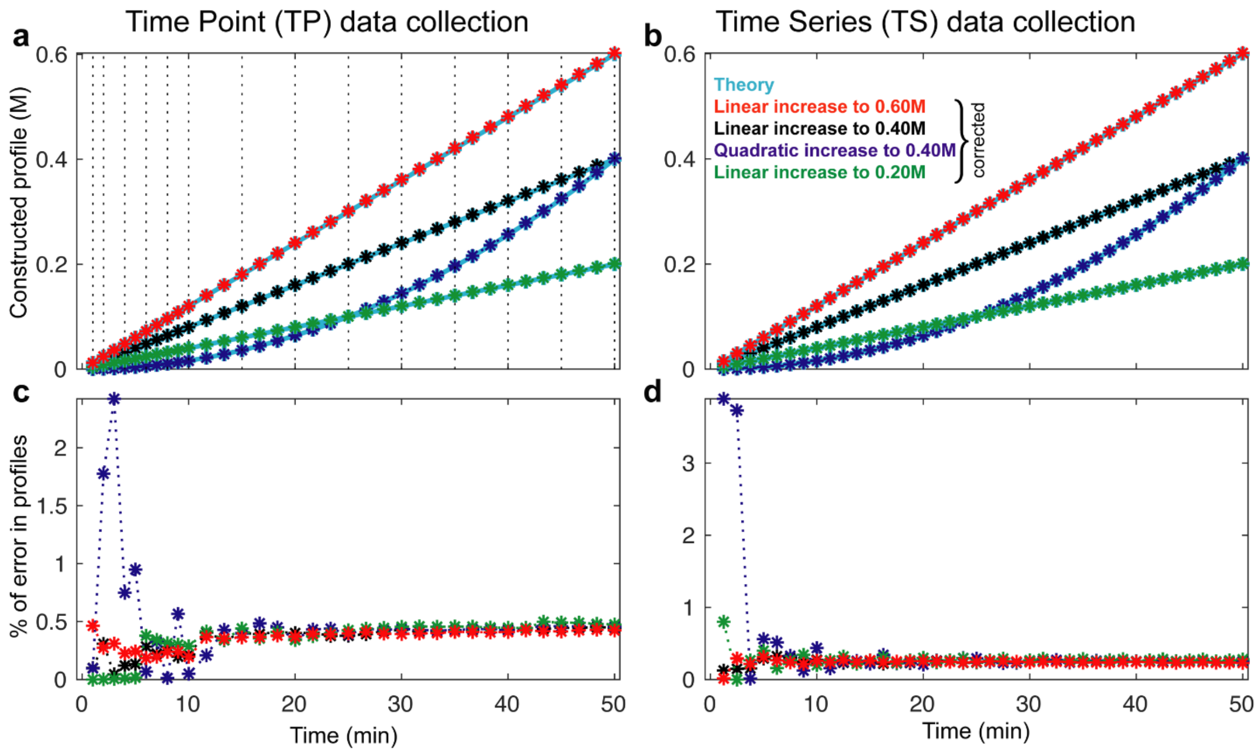

Supplementary Figure 2 | Calculated pump profiles for increasing linear and nonlinear gradient kinetics. (**a-b**) Calculation of pump profile generation for Time Point (TP, **a**) and Time series (TS, **b**) data collection. In both TP and TS experiments, computed pump profiles (corrected for volume removal and therefore change in stimulus concentration) are linear increasing gradient to 0.60M (red), linear increasing gradient to 0.40M (black), quadratic increasing gradient to 0.40M (blue), and linear increasing gradient to 0.20M (green), all compared to their theoretical values (cyan). (**c-d**) Error comparisons between computed pump profiles and their corresponding proposed concentration profiles for TP (**c**) and TS (**d**) experiments. The profiles are generated under the following conditions (the same conditions are used for **Supplementary Figure 3**); the concentrated stimulus concentration $C_{max} = 4\ M$. The total flask volume is set $V_0 = 50\ mL$ at $\mathrm{t} = 0$. Pump 2 rate was set to $\bar{k} = 0.1\ mL/min$ for TS and $\bar{k} = 0$ for TP experiment. Samples are taken out at the fixed volumes of $dw_i = 1\ mL$ at the time points [1,2,4,6,8,10,15,20,25,30,35,40,45,50] minutes for TP (dotted lines in **a**), while no sampling is done for TS. Both TP and TS profiles are generated over 50 minutes. TS is computed in 40 intervals and TP profile in 34 intervals set optimally by the programmable syringe pump.

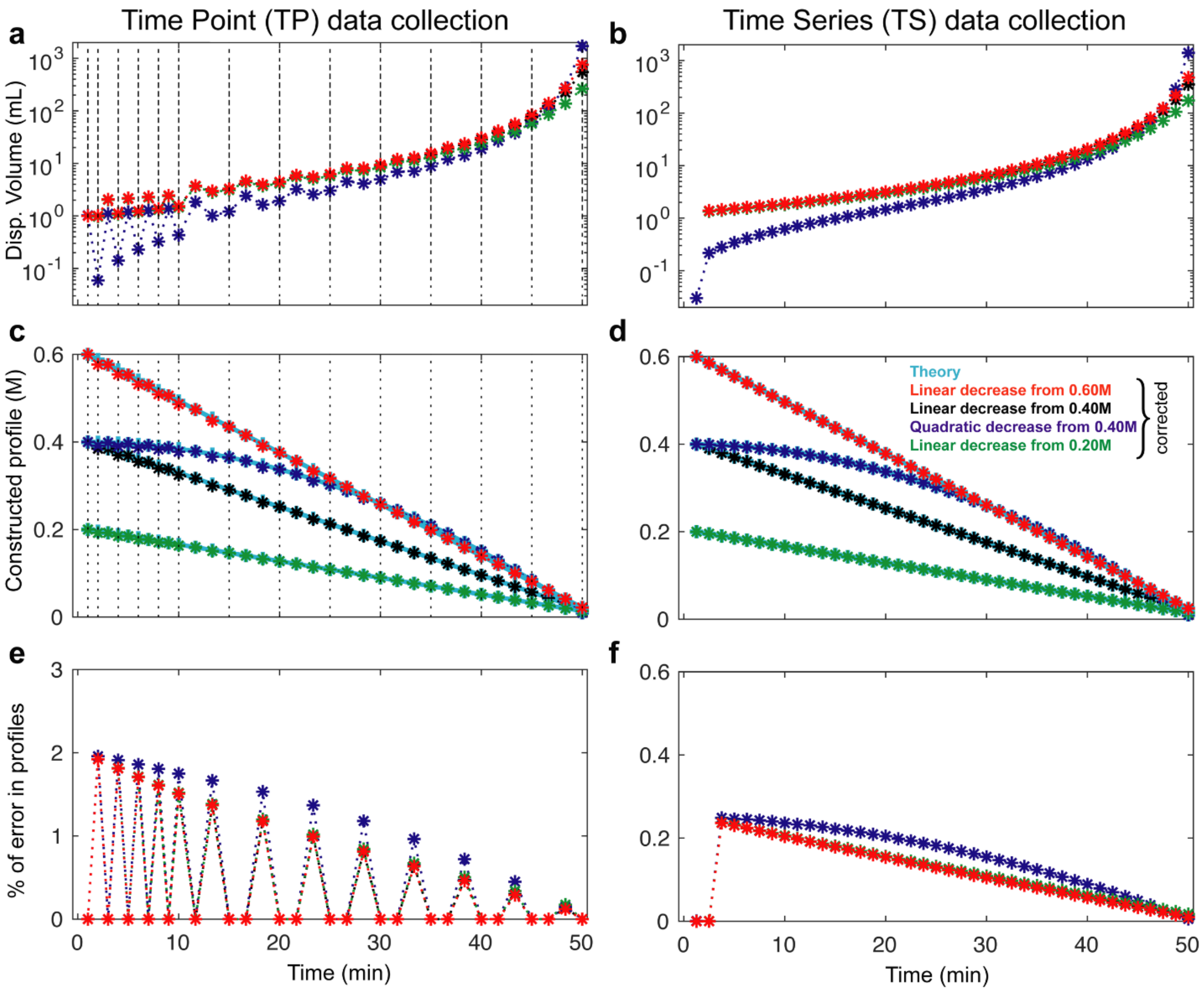

Supplementary Figure 3 | Calculated pump profiles for decreasing linear and nonlinear gradient kinetics. (**a-f**) Calculation of decreasing pump profile generation for Time Point (TP, **a,c,e**) and Time series (TS, **b,d,f**) data collection. In both TP and TS experiments, computed pump profiles (corrected for volume removal and therefore change in stimulus concentration) are linear decreasing gradient from 0.60 M (red), linear decreasing gradient from 0.40 M (black), quadratic decreasing gradient from 0.40 M (blue), and linear decreasing gradient from 0.20 M (green), all to 0.01M and all compared to their theoretical values (cyan). (**a-b**) Computed and instrument adapted syringe dispense volume. (**c-d**) Computed concentration profiles over time. (**e-f**) Error comparisons between computed decreasing pump profiles and their corresponding proposed concentration profiles for TP (**e**) and TS (**f**) experiments.

Table S1. Calculation results for TS experiment profile generation.

Interval	Time points (min)	Disp. Volume* (mL)	Cumulative Disp. Volume (mL)	Pump rate (μL /min)	Molarity* (M)	Error* % in molarity compared to theory
1	1.25	0.125	0.125	100	0.01	0
2	2.5	0.126	0.251	100.8	0.02	0
3	3.75	0.126	0.377	100.8	0.03	0
4	5	0.127	0.504	101.6	0.04	0
5	6.25	0.127	0.631	101.6	0.05	0
6	7.5	0.127	0.758	101.6	0.06	0
7	8.75	0.127	0.885	101.6	0.07	0
8	10	0.128	1.013	102.4	0.08	0
9	11.25	0.128	1.141	102.4	0.09	0
10	12.5	0.128	1.269	102.4	0.1	0
11	13.75	0.129	1.398	103.2	0.11	0
12	15	0.129	1.527	103.2	0.12	0
13	16.25	0.129	1.656	103.2	0.13	0
14	17.5	0.13	1.786	104	0.14	0
15	18.75	0.13	1.916	104	0.15	0
16	20	0.131	2.047	104.8	0.16	0
17	21.25	0.131	2.178	104.8	0.17	0
18	22.5	0.131	2.309	104.8	0.18	0
19	23.75	0.131	2.44	104.8	0.19	0

20	25	0.132	2.572	105.6	0.2	0
21	26.25	0.133	2.705	106.4	0.211	0.476
22	27.5	0.132	2.837	105.6	0.221	0.455
23	28.75	0.133	2.97	106.4	0.231	0.435
24	30	0.134	3.104	107.2	0.241	0.417
25	31.25	0.134	3.238	107.2	0.251	0.4
26	32.5	0.134	3.372	107.2	0.261	0.385
27	33.75	0.134	3.506	107.2	0.271	0.37
28	35	0.135	3.641	108	0.281	0.357
29	36.25	0.135	3.776	108	0.291	0.345
30	37.5	0.136	3.912	108.8	0.301	0.333
31	38.75	0.136	4.048	108.8	0.311	0.323
32	40	0.137	4.185	109.6	0.321	0.312
33	41.25	0.137	4.322	109.6	0.331	0.303
34	42.5	0.137	4.459	109.6	0.341	0.294
35	43.75	0.138	4.597	110.4	0.351	0.286
36	45	0.138	4.735	110.4	0.361	0.278
37	46.25	0.138	4.873	110.4	0.371	0.27
38	47.5	0.139	5.012	111.2	0.381	0.263
39	48.75	0.14	5.152	112	0.391	0.256
40	50	0.14	5.292	112	0.401	0.25

Table S2. Calculation results for TP experiment profile generation.

Interval	Time points (min)	Disp. Volume* (mL)	Cumulative Disp. Volume (mL)	Pump rate (μL/min)	Molarity* (M)	Error* % in molarity compared to theory
1	1	0.1	0.1	100	0.008	0
2	2	0.099	0.199	99	0.016	0
3	3	0.097	0.296	97	0.024	0
4	4	0.097	0.393	97	0.032	0
5	5	0.096	0.489	96	0.04	0
6	6	0.096	0.585	96	0.048	0
7	7	0.095	0.68	95	0.056	0
8	8	0.094	0.774	94	0.064	0
9	9	0.094	0.868	94	0.072	0
10	10	0.093	0.961	93	0.08	0
11	11.667	0.154	1.115	92.4	0.093	0.357
12	13.333	0.154	1.269	92.4	0.107	0.312
13	15	0.156	1.425	93.6	0.12	0
14	16.667	0.153	1.578	91.8	0.133	0.25
15	18.333	0.154	1.732	92.4	0.147	0.227
16	20	0.156	1.888	93.6	0.16	0
17	21.667	0.152	2.04	91.2	0.173	0.192
18	23.333	0.154	2.194	92.4	0.187	0.179
19	25	0.155	2.349	93	0.2	0

20	26.667	0.153	2.502	91.8	0.213	0.156
21	28.333	0.154	2.656	92.4	0.227	0.147
22	30	0.155	2.811	93	0.24	0
23	31.667	0.152	2.963	91.2	0.253	0.132
24	33.333	0.153	3.116	91.8	0.267	0.125
25	35	0.155	3.271	93	0.28	0
26	36.667	0.152	3.423	91.2	0.293	0.114
27	38.333	0.153	3.576	91.8	0.307	0.109
28	40	0.154	3.73	92.4	0.32	0
29	41.667	0.152	3.882	91.2	0.333	0.1
30	43.333	0.153	4.035	91.8	0.347	0.096
31	45	0.154	4.189	92.4	0.36	0
32	46.667	0.151	4.34	90.6	0.373	0.089
33	48.333	0.153	4.493	91.8	0.387	0.086
34	50	0.154	4.647	92.4	0.4	0

*Note on rounding the values: We round the calculated values of dv_i (in mL) to 3 decimal places, which is required by the software of the syringe pump. The resulting calculated pump rates for k_i (in μL /min) are within the range of recommended minimum to maximum pump rate of the syringe pump. The reconstructed molarities and their errors are plotted without rounding in Figure 3 and **Supplementary Figures 2-3**, while in Tables 1 and 2 we round them to 3 decimal places for illustration purposes.
